# A novel mutation in the PRPH2 gene in a Chinese pedigree with retinitis pigmentosa and angle-closure glaucoma

**DOI:** 10.1186/s12886-021-02064-5

**Published:** 2021-08-16

**Authors:** Wei-ning Li, Xiu-juan Du, Yu-ting Zhang, Le-yi Wang, Jing Zhu

**Affiliations:** 1 Department of Ophthalmology, Qilu Hospital, Cheeloo College of Medicine, Shandong University, 107#, Wenhua Xi Road, Jinan, Shandong 250012 People’s Republic of China; 2 Affiliated Eye Hospital of Shandong University of TCM, Jinan, Shandong 250002 People’s Republic of China

**Keywords:** Retinitis pigmentosa (RP), Angle-closure glaucoma (ACG), Peripherin-2 (PRPH2), Whole-exome sequencing (WES)

## Abstract

**Background:**

Retinitis pigmentosa (RP) is a rare, progressive, and hereditary disorder that leads to the progressive loss of vision and visual field, and in some cases blindness. The specific relationship between RP and glaucoma has been debated for decades.

**Methods:**

In this study, we examined a Han RP family with concomitant angle-closure glaucoma (ACG), performed an inductive analysis of their clinical features and assistant results, and applied whole-exome sequencing (WES) technology for a molecular diagnosis.

**Results:**

A novel transversion mutation (c.626 T > A) was identified in the peripherin-2 (PRPH2) gene in the proband, resulting in the substitution of Valine to aspartic acid in codon 209. A full ophthalmic examination showed that the proband with the c.626 T > A mutation had a typical RP manifestation, with close angles; however, the proband’s elder brother, who lacked the novel mutation, had a normal fundus and open angles.

**Conclusion:**

Our results extend the genetic mutation spectrum of PRPH2 in RP, and provide evidence to support a genetic correlation between RP and ACG.

## Background

Retinitis pigmentosa (RP) is a rare, progressive, hereditary, and dystrophic degenerative disorder, which impairs the function of photoreceptors and the retinal pigment epithelium, and leads to the progressive loss of vision and visual field, and even blindness [1]. Since Galezowski first reported a case of RP associated with glaucoma in 1862 [2], the relationship between RP and glaucoma has been debated upon. Badeeb O reported that the prevalence of primary open-angle glaucoma with RP ranged from 2 to 12%, and that the incidence of primary angle-closure glaucoma (ACG) was 1.03% in RP patients over 40 years of age [3]. Ko YC’s research showed that RP patients have a 3.64-fold greater risk of having ACG, than individuals without RP in Taiwan [4]. However, Xu J ‘s study demonstrated that RP patients with ACG shared similar biometric characteristics with single ACG patients, and suggested that the association between RP and ACG might be coincidental [5]. Ultimately, the existence of a relationship between RP and ACG requires further study.

It is well known that the peripherin-2 (PRPH2) gene (NM_000322.4) encodes a photoreceptor specific transmembrane glycoprotein with 346 amino acids (also known as retinal degeneration slow or RDS), which is involved in the formation of the photoreceptor outer segment [6, 7]. Mutations in the PRPH2 gene result in degeneration in both central and peripheral retina, and lead to a variety of retinal degenerative diseases, such as RP [6], macular and cone/cone-rod dystrophies [8], foveomacular vitelliform dystrophy [9], central areolar choroidal dystrophy, and other forms of late-onset macular degeneration [10]. However, the potential association between PRPH2 and glaucoma has not been reported in the literature.

In this study, we focused on a Han RP family with concomitant ACG, and analyzed the patient’s clinical manifestation and ophthalmic examination results in detail and applied whole-exome sequencing and Sanger sequencing technology to the proband. A novel transversion mutation (c.626 T > A) in PRPH2 was found in the proband. Our results provide evidence to support the correlation between RP and ACG, and contribute to advancements in genetic counseling.

## Materials and methods

### Proband, pedigree and clinical data

**Fig. 1:**
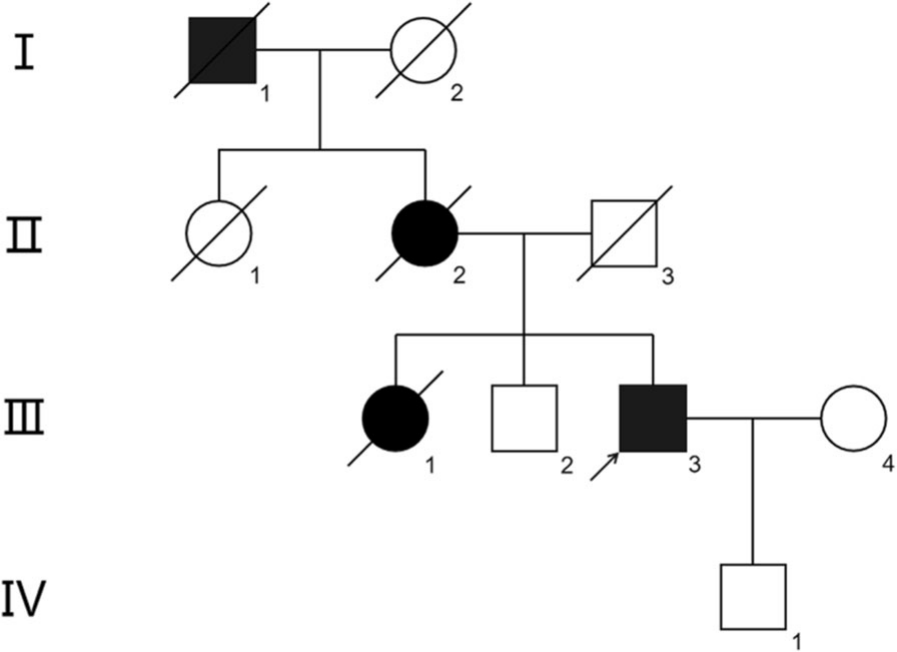
Pedigree of mainland Chinese Han family with inherited retinal degenerative disease. An autosomal dominant inheritance pattern is shown. Closed and open symbols indicate affected patients and unaffected subjects, respectively. The arrow indicates proband. A slash indicates that the individual is deceased

The proband (Fig. 1, III: 3) was a 60 years old Han male and presented to us with blurring of vision and recurrent short episodes of pain in the right eye for a period of more than 1 year. The intraocular pressure (IOP) was 31 mmHg oculus dexter (OD) and 16 mmHg oculus sinister (OS), and the anterior chamber of both eyes was shallow with closed angles. Based on this, the patient was initially diagnosed with chronic angle closure glaucoma. During genetic counseling, the patient revealed that his maternal grandfather, mother, elder sister, and himself all suffered with nyctalopia at an early age. The patient and his sister received a clinical diagnosis of RP, and his sister underwent glaucoma surgery, when she was alive. The patient’s elder brother, who was 65 years old at the time of genetic counseling, was considered to be completely healthy, without any clinical indications of RP. The proband underwent a full ophthalmic examination, including fundus photography, visual field testing, optical coherence tomography (OCT), and ultrasound biomicroscopy (UBM). Peripheral blood samples were collected from all participants for genomic DNA extraction using standard protocols [11].

### Whole-exome sequencing

Whole-exome sequencing (WES) was performed on DNA from peripheral blood. After the fragmentation of genomic DNA, ligation of the paired-End adapter, and amplification and purification, all exons and 50 bp regions adjacent to introns were captured using the SeqCap EZ Med Exome Enrichment Kit (Roche NimbleGen). The DNA library was prepared by post-capture amplification and purification, followed by sequencing with the Illumina HiSeq sequencing platform. Sequence data alignments to the human genome reference (hg19) and variant-calling were performed using NextGene V2.3.4 software, further to get the coverage and mean read depth of the target regions. The mean read depth was 249.70×, and read depth reached 20× for 97.202% of the target sequences [12]. Variants were screened as followed: 1) Preference to the variants related to the diseases, small INDEL, canonical splice sites and missense variants. 2) Minor allele frequency in normal populations < 5% (except for the known MAF > =5% pathogenicity). 3) Preference to the variants in HGMD, ClinVar. 4) Preference to the variants in OMIM. The variants of pathogenicity were according to Standards and guidelines for the interpretation of sequence variants published by ACMG in 2015 with HGVS nomenclature.

### Sanger sequencing

Sanger sequencing was performed to verify the mutation identified in the PRPH2 gene. The following primers were used for PCR amplifications: PRPH2 forward 5’to 3′:TGTCTTCAGCGCCTAGAACAGTGA; and PRPH2 reverse 5’to 3′:AAGGCTGTTTCCAAAGAGGGAGG.

### Bioinformatics analysis

Analysis including the conservation of nucleotide bases and amino acids, the frequency of the normal population (1000 Genomes Project, ExAC, dbSNP database and locus specific databases), as well as the use of data from Human Gene Mutation Database (HGMD), ClinVar database, and Online Mendelian Inheritance in Man (OMIM), were performed by NextGene V2.3.4 software and lab’s own scripts [13]. The potential deleterious effects and biological function of the mutation were predicted using SIFT (http://sift.jcvi.org), PolyPhen-2 (http://genetics.bwh.harvard.edu/pph2/), and Mutation Taster (http://www.mutationtaster.org/). The variance of pathogenicity was evaluated according to the Standards and Guidelines for the Interpretation of Sequence Variants, published by American College of Medical Genetics (ACMG) in 2015 with HGVS nomenclature. I-TASSER software was used to model and PyMOL Viewer was used to visualize the protein configuration prediction results.

## Results

### Proband and clinical characteristics

**Fig. 2:**
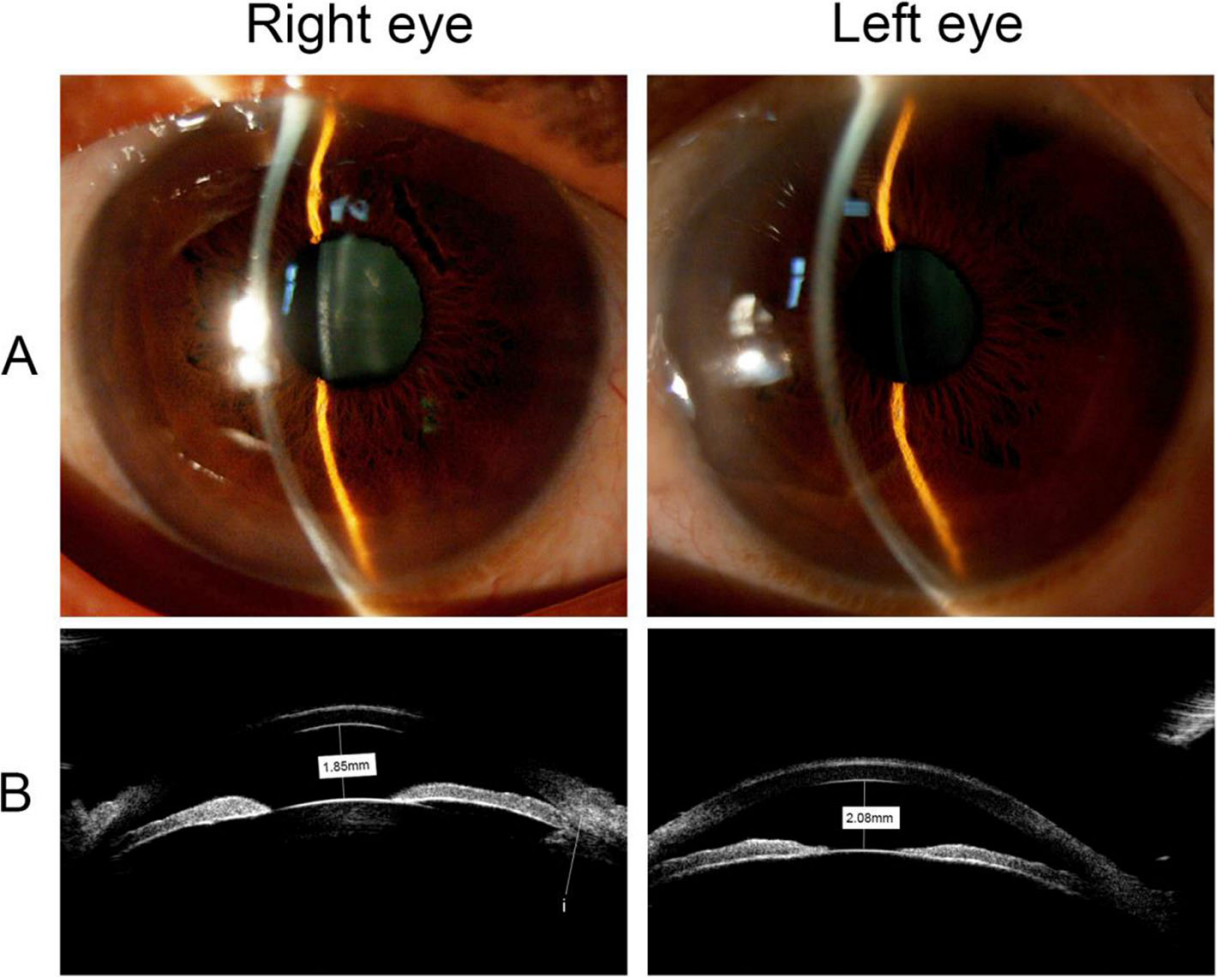
The anterior segment sign of the proband’s eye. **A** the slit-lamp microscope showed a shallow anterior chamber in both eyes of the proband. **B** the UBM examination showed that the depth of the anterior chamber was 1.85 mm OD and 2.08 mm OS, with closed angles (i) in both eyes

The pedigree of this family showed an autosomal dominant form of RP (Fig. 1). Physical examination of the proband (III: 3) excluded systemic disorders. Upon ophthalmic examination, uncorrected visual acuity was 20/500 OD and 20/133 OS. Corrected distance visual acuity (CDVA) was 20/200 with a refraction of − 3.50-1.25 × 011 OD, and 20/66 with a refraction of − 2.25-1.50 × 170 OS. The shallow anterior chamber (1.85 mm OD and 2.08 mm OS) with closed angles in both eyes was confirmed by both slit-lamp microscope and UBM (Fig. 2a, b). These results are in accordance with the clinical manifestations of closure glaucoma.

**Fig. 3:**
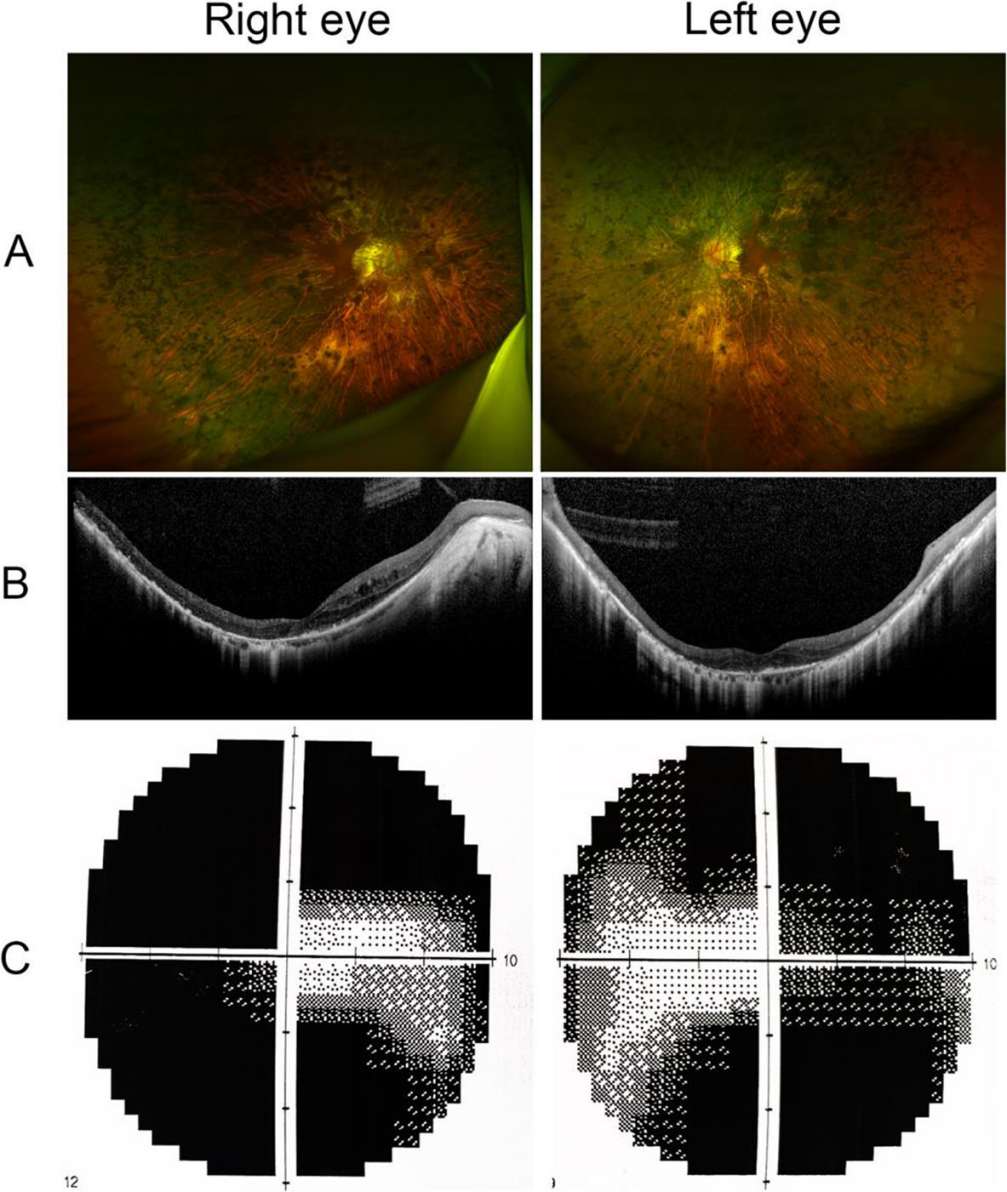
The supplementary examinations of the proband’s eye. **A** The images of fundus changes in the proband. **B** The OCT examination showing the destroyed structure of IS/OS layer. **C** Visual field examination showing severe visual field constriction for the proband

The patient also presented characteristic fundus features of RP, including the waxy pallor optic disc, attenuated retinal vessels, and bone spicule pigment deposits (Fig. 3a). OCT revealed that the continuity of the IS/OS (Inner Segment/Out Segment) layer was destroyed, demonstrating degenerative changes of the photoreceptor cell (Fig. 3b). Tubular visual field was observed in both eyes during visual field testing (Fig. 3c). The results of examination using the slit-lamp microscope, gonioscope, as well as the ophthalmoscope examination of the proband’s elder brother and son, were normal (data not shown). Because of the unsatisfactory effect of antiglaucomatous drugs, the proband underwent trabecular filtration surgery for both eyes, and after a one-year follow-up the vision and visual field was found to be stable for both eyes.

### Detection of mutations

**Fig. 4:**
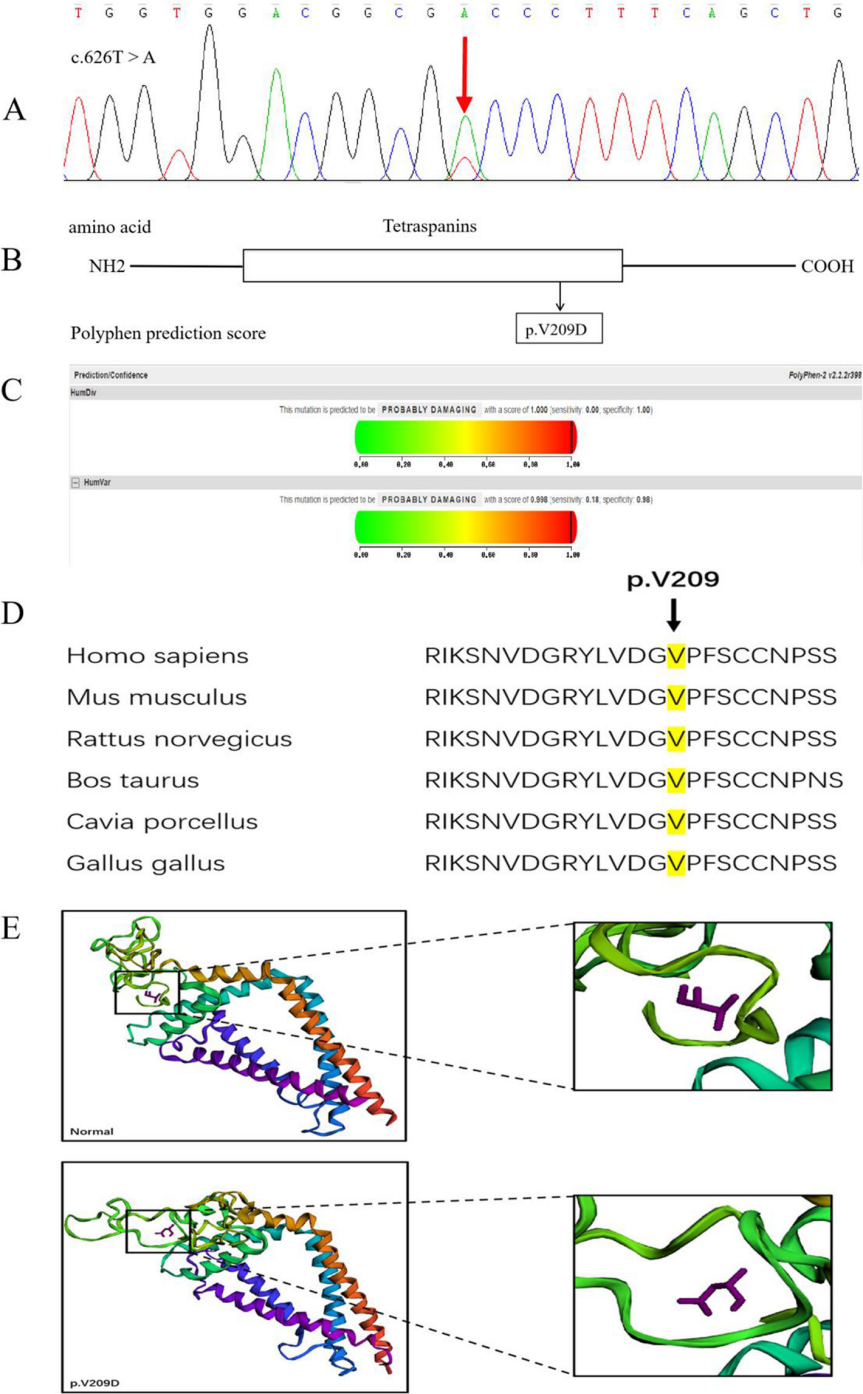
Sequencing results and bioinformatic analysis of the gene mutation in our study. **A** Partial sequence diagram of PRPH2 exon 2. A heterozygous mutation c.626 T > A transition, causing the substitution of Valine to aspartic acid in codon 209, is shown with an arrow. **B** The structural domains of PRPH2. Mutations at the protein level are indicated below the domains. **C** Score of the novel damaging mutation c.626 T > A (p.V209D) in Polyphen v.2. **D** Cross-species conservation of PRPH2 in the vicinity of the mutation (p.V209) is displayed. **E** Protein structure prediction results of wild-type and mutant PRPH2

To validate the diagnoses, we applied WES technology to the proband and his family members. Sequence data alignments to the human genome reference (hg19) and variants-calling were used by NextGene V2.3.4 software, further to get the coverage and mean read depth of the target regions. The mean read depth was 249.70×, and read depth reached 20× for 97.202% of the target sequences [12]. Based on current public databases, non-pathogenic variations was filtered out with IFT, Polyphen 2 and MutationTaster and were prioritized for further confirmation and characterization. A single nucleotide heterozygous, transversion mutation (c.626 T > A) of exon 2 in the PRPH2 gene in the proband was identified, resulting in the substitution of a Valine to an aspartic acid in codon 209 (Fig. 4a). The substitution was not detected in 1000 Genomes Project, indicating that it was not a polymorphism. Human PRPH2 has a tetraspanin domain, which is a transmembrane receptor glycoprotein with 4 transmembrane domains. The transversion mutation was found in the tetraspanin domain at position 209 (V209D) (Fig. 4b). Moreover, this mutation was predicted to be damaging using both Polyphen v.2, with a score of 0.998, and SIFT software programs (Fig. 4c). MutationTaster deduced that this mutation had a high probability of affecting protein properties, and was pathogenic. The conservation of p.V209 in various species was demonstrated in multiple amino acid sequence alignments, using the ClustalW tool (Fig. 4d). This novel mutation in PRPH2 is predicted to cause an abnormal connection of nascent outer segment discs, leading to defects in the formation of the photoreceptor outer segment (Fig. 4e). Other variants in genes for IFT172, DFNB31, PDHX, SALL2, BMP4 and GPR179 by WES were excluded as deleterious and pathogenic mutations, and none of them was reported as a stronger/convincing glaucoma causing variant in the literature.

## Discussion

The human PRPH2 gene is mapped to chromosome 6 and encodes a glycoprotein which includes both N and C cytoplasmic termini, four transmembrane domains, and two asymmetric extra cytosolic domains called intradiscal loop 1 and 2 [14]. The PRPH2 tetraspanin domain is involved in the process which converts a chemical phototransduction signal into an electrical one in the outer segment of the retina, so alterations of PRPH2 structure could lead to impairment of vision function, with varying degrees [15]. Previous studies have shown that the majority of mutations in PRPH2 mainly occur in intradiscal loop 2 [16, 17] In our patient, the identified c.626 T > A transversion mutation resulted in the substitution of a Valine to an aspartic acid in codon 209, which is also located in intradiscal loop 2 of exon 2. This mutation has been only reported by Birtel J, in two patients with macular dystrophies; however, no related literature was found for RP patients [8]. The deletion of codons 206–209 caused a larger in-frame deletion, and resulted in autosomal dominant RP [17]. And the deletion of codons 203–209 has been reported to lead to macular degeneration [18] In 15 RP cases reported by Keen, 8 cases involved mutations in codons 210–216 [17]. Based on HGMD (http://www.hgmd.cf.ac.uk/ac/index.php), approximately 15% of reported mutations in PRPH2 involve codons 203–216. Therefore, we inferred that this region might be particularly significant for the role of PRPH2 in rods, or the peripheral retina.

Recently, researchers have demonstrated a relationship between some retinal dystrophy-causing genes and glaucoma [19]. Fernandez-Martinez L showed that the RPGRIP1 gene, which was known as retinitis pigmentosa GTPase regulator-interacting protein 1, was considered as a risk factor for primary open angle glaucoma, and that variants of RPGRIP1 might increase an individual’s susceptibility to various forms of glaucoma [20]. Micheal S reported that PRPF8 mutations were associated with both autosomal dominant RP, and adult-onset POAG [21]. RetNet genes also appear to be associated with a significant proportion of PACG, especially in probands with both PACG and RP [19]. However, the genetic correlation between PRPH2 and glaucoma has not been reported yet. In this study, the proband with the PRPH2 c.626 T > A mutation showed a typical RP manifestation with close angles, yet the proband’s elder brother, lacking this novel the mutation, had a normal fundus and open angles. Therefore, we inferred that ACG was an accompanying symptom of RP, in this case, and that the PRPH2 mutation might be related to the co-occurrence of glaucoma and RP.

Based on the hereditary nature of RP and current research regarding RP and ACG, ACG is not considered as a risk factor of RP, however, some RP associated ocular manifestations, such as zonular insufficiency, nanophthalmos, or ectopic lentis, might explain the increased prevalence of ACG in RP patients [4]. Badeeb et al’s study showed that a thicker and more anteriorly positioned lens could be observed in RP patients with normal axial length [3]. Several studies have shown that zonular instability was common in RP patients, which might lead to the anterior displacement of the lens [22]. All of the above manifestations could result in subsequent shallow anterior chamber and angle narrowing, and even angle closure [23]. In this study, although no lenticular tremor was observed in the proband, the abnormal shallow anterior chamber indicated the probability of the existence of zonular instability.

It is well known that a high IOP in RP patients aggravates damage of the optic nerve and defects of the visual field, resulting in devastating visual impairment in the short term [24]. Thus far, IOP control is the main treatment option for this condition. In this study, due to the unsatisfactory effect of antiglaucomatous drugs, we chose trabecular filtration surgery for the proband, and after a one-year follow-up the vision and visual field was found to be stable. However, some research has shown that the pathogenesis of both RP and glaucoma involves the immune system. Massengill MT reported that a chronic inflammatory process, mediated by Müller glial and microglial cells, could be observed in RP [25]. Ten’s research showed that the expression of IL-2, IL-6, monocyte chemoattractant protein-1 (MCP-1), and placental growth factor (PlGF) were significantly up-regulated in the intraocular fluid of RP patients, and the level of IL-8 was higher in presence of glaucoma [26]. Serious or chronic inflammation might influence the function of the trabecular meshwork and lead to an increase in IOP and damage to the optic nerve [27]. Thus, immunosuppression and neuroprotection might represent potential therapeutic targets for RP and glaucoma in the future.

## Conclusions

In this study, we examined a Han RP family with concomitant ACG based on clinical data, ophthalmic examination, and genetic testing. Our results extend the genetic mutation spectrum of PRPH2 in RP, and provide evidence to support a correlation between RP and ACG. However, the identification of a relationship between the phenotype and genotype, requires additional studies in more patients.

## Data Availability

The datasets used and/or analysed during the current study are available from the corresponding author on reasonable request.

## Abbreviations

RP: Retinitis pigmentosa
ACG: Angle-closure glaucoma
PRPH2: Peripherin-2
IOP: Intraocular pressure
OD: Oculus dexter
OS: Oculus sinister
OCT: Optical coherence tomography
UBM: Ultrasound biomicroscopy
WES: Whole-exome sequencing
HGMD: Human Gene Mutation Database
OMIM: Online Mendelian Inheritance in Man
IS/OS: Inner Segment/put Segment
